# Grade III Severe QT Prolongation in an Indian Male on All-Oral Longer Regimen for Multidrug-Resistant Pulmonary Tuberculosis: World’s First Case

**DOI:** 10.7759/cureus.31819

**Published:** 2022-11-23

**Authors:** Sankalp Yadav

**Affiliations:** 1 Medicine, Shri Madan Lal Khurana Chest Clinic, Moti Nagar, New Delhi, IND

**Keywords:** multidrug-resistant (mdr) tb, moxifloxacin, prolonged qtc interval, qt interval, tuberculosis

## Abstract

Antitubercular drugs are associated with several adverse drug reactions (ADRs). Some of these ADRs are life-threatening and require immediate attention and hospital admission. With the development of new regimens and inclusions of newer drugs such as bedaquiline, pretomanid, and delamanid, it is imperative to have an eye for the side effects. A number of antitubercular drugs such as bedaquiline, moxifloxacin, clofazimine, pretomanid, and delamanid are known to cause ADRs on the heart. Herein, a case of grade III severe QT prolongation with corrected QT (QTc) of 688 ms in an Indian male on a WHO-recommended all-oral longer regimen (AOLR) for multidrug-resistant (MDR) pulmonary tuberculosis (TB) is presented. This episode happened on the sixth day post his treatment initiation, thereby making it the earliest of such findings. The patient was managed conservatively, and his baseline electrocardiogram (ECG) returned to normal with QTc of 432 ms with the offending drug as moxifloxacin, which was omitted from the regimen and replaced with delamanid. There are some cases similar to this case available in the literature; however, grade III severe QT prolongation with QTc of 688 ms in a male on a WHO-recommended all-oral longer regimen for multidrug-resistant pulmonary tuberculosis is never reported.

## Introduction

Tuberculosis (TB) is a disease known to humankind for a long and is caused by the bacteria *Mycobacterium tuberculosis *[1]. TB is a critical public health issue and is a substantial contributor to morbidity and mortality, especially in the high-burden countries of Africa, Asia, and Europe [2]. The global TB report by the WHO for the year 2022 estimates that nearly 10.6 million people caught the infection in the year 2021, which was equivalent to 134 cases (95% uncertainty interval {UI}: 125-143) per 0.1 million population [3]. The geographical distribution of these cases is Southeast Asia, 45%; Africa, 23%; the Western Pacific, 18%; Eastern Mediterranean, 8.1%; the Americas, 2.9%; and Europe, 2.2% [3].

Drug-resistant TB (DR-TB) continues to be a challenge for TB control programs, and the same is evident in an increase of 3.1% with 450,000 incident cases (95% uncertainty interval {UI}: 399,000-501,000) of multidrug-resistant (MDR)/rifampicin-resistant (RR) TB in 2021 as compared to 2020 [3]. Further, this high morbidity resulted in about 191,000 (range: 119,000-264,000) deaths due to multidrug-resistant or rifampicin-resistant tuberculosis (MDR/RR-TB) in 2021 [3].

The recent developments in the management of TB include the addition of newer drugs such as bedaquiline, delamanid, and pretomanid [4,5]. Not only the inclusion of drugs but also the implementation of several newer regimens for the fight against TB have been included in the national TB control programs [6]. As evidenced by the published data, antitubercular drugs are associated with adverse drug reactions (ADRs) [7]. The situation becomes grave when the ADRs are life-threatening [7]. Here, a case of ADR due to moxifloxacin on the QT interval is reported in an Indian male who was initiated on the all-oral longer regimen (AOLR) for pulmonary MDR-TB. However, other drugs such as bedaquiline and clofazimine were also a part of his treatment, and therefore, it was a challenging task to identify the offending drug. A detailed literature review of similar cases showed that a case with grade III severe QT prolongation with corrected QT (QTc) of 688 ms in a HIV-nonreactive male on a WHO-recommended AOLR for pulmonary MDR-TB has never been reported in the literature.

## Case presentation

A 38-year-old Indian male laborer by profession with a history of being treated for drug-sensitive tubercular left-sided cervical lymphadenitis (168 days) came to the outpatient department with chief complaints of fever for one week, cough with expectoration for two weeks, and loss of appetite and weight for two weeks. He was all right two weeks ago when he developed a cough that was continuous and associated with greenish-colored non-foul-smelling expectoration and was relieved for some time after he took an antitussive (syrup ambroxol). He developed a fever about a week ago, which was evening rise not associated with chills or rigor and subsided with over-the-counter antipyretic paracetamol. He also complained of loss of appetite with a loss of around 3 kg of weight in two weeks. Further, there was no history of hemoptysis, chest pain, seizures, or COVID-19 to him or any of his contacts.

General examination was suggestive of a lean male with a weight of 43 kg, pulse of 88/minute, blood pressure of 110/80 mm Hg, temperature of 99 degrees Fahrenheit, respiratory rate of 19/minute, and oxygen saturation (SpO_2_) of 96% on room air. There was no edema, pallor, cyanosis, lymphadenopathy, or icterus. Systemic examination was remarkable for a dull note, which was heard on bilateral upper lobes, and auscultation revealed reduced breath sounds in the left and right upper zones of the chest. The rest of the systemic examinations were within normal limits.

**Figure 1:**
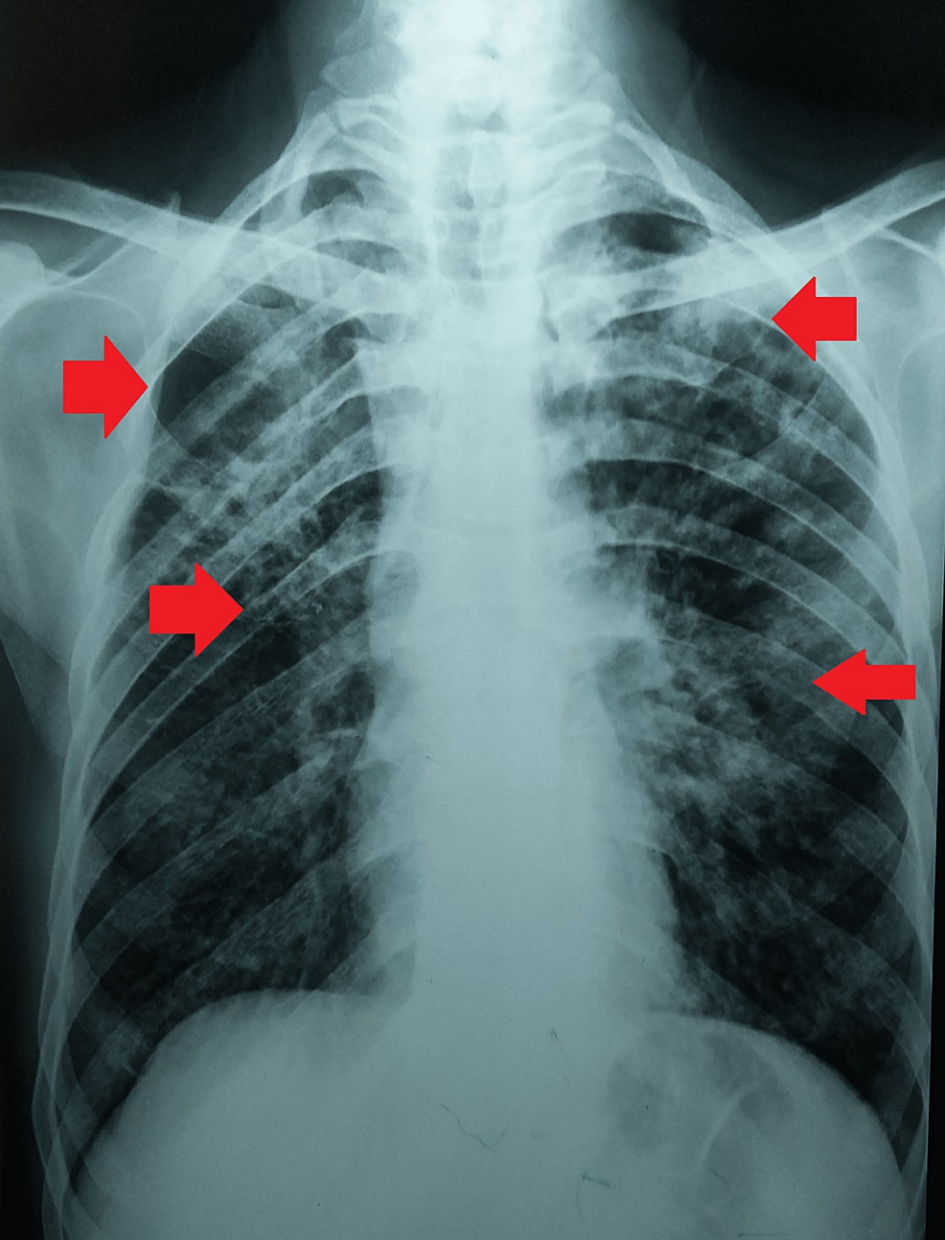
Chest radiograph (PA view) was suggestive of bilateral opacities with a cavity on the right upper lobe PA: posteroanterior

The initial workup involved a chest radiograph (posteroanterior {PA} view), sputum microscopy for acid-fast bacilli (AFB), cartridge-based nucleic acid amplification test (CBNAAT), and complete blood count (CBC). The chest radiograph (PA view) was suggestive of bilateral opacities with a cavity on the right upper lobe (Figure 1). The fluorescent microscopy of sputum for AFB was positive (3+), and the same was evident from the report of CBNAAT, which was *Mycobacterium tuberculosis* detected (high) with rifampicin resistance. CBC was remarkable for raised erythrocyte sedimentation rate of 80 mm in the first hour.

Based on the reports, he was diagnosed as a pulmonary multidrug-resistant (MDR) TB case, and he was referred to the nodal DR-TB center for pretreatment evaluation (PTE) to start the AOLR for MDR-TB. As per the programmatic management of drug-resistant tuberculosis (PMDT) guidelines, his samples for line probe assay (LPA), culture, and drug susceptibility testing (DST) were sent to the intermediate reference laboratory (IRL) [6]. His PTE was within normal limits with a baseline electrocardiogram (ECG) showing QTc of 410 ms. As per his weight, his regimen was finalized as mentioned in Table 1.

**Table 1:** All-oral longer regimen for multidrug-resistant pulmonary tuberculosis

Drugs (Tablets)	Doses (Per Oral)
Bedaquiline	400 mg daily for two weeks and then 200 mg alternate daily for 22 weeks
Moxifloxacin (high dose)	600 mg daily
Linezolid	600 mg daily
Clofazimine	100 mg daily
Cycloserine	500 mg daily
Pyridoxine	100 mg daily

**Figure 2:**
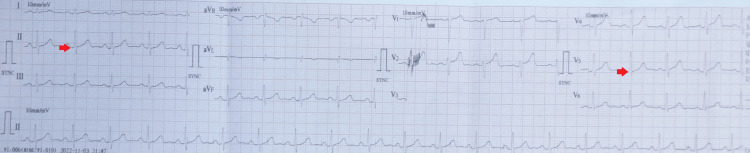
Electrocardiogram (ECG) showing prolongation of QT interval

After six days post starting the AOLR, during a routine check-up, he underwent an ECG. The ECG was abnormal with QTc of 688 ms and QTc using the Fridericia formula (QTcF) of 638 ms (Figure 2).

**Figure 3:**
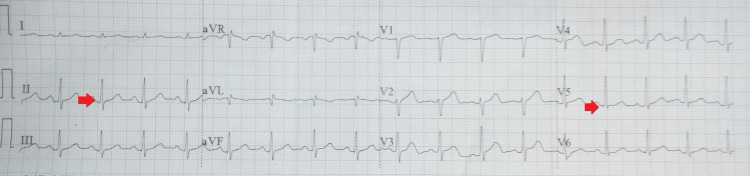
A normal electrocardiogram (ECG) with QTc of 432 ms QTc: corrected QT

Other investigations such as serum electrolytes, CBC, liver function tests, and renal function tests were within normal limits. All the medications were stopped, and he was referred to the nodal DR-TB center where he was admitted and managed conservatively. He had no complaints and was asymptomatic. Vitals were pulse of 89/minute, blood pressure of 120/70 mm Hg, temperature of 98.4 degrees Fahrenheit, respiratory rate of 22/minute, and SpO_2_ of 97% on room air. Repeat ECG 12 hours apart was indicative of QTc of 538 ms. Another ECG (24 hours apart) showed that the QTc was 430 ms. All the drugs were started in a stepwise manner as per the PMDT guidelines with strict ECG monitoring [6]. There was no abnormality in the QT interval until high-dose moxifloxacin was initiated. Post moxifloxacin initiation, there was a prolongation of QT interval with QTc of 510 ms. Therefore, the QT interval prolongation was attributed to moxifloxacin, and the same was stopped and replaced with delamanid 100 mg twice a day for 24 weeks. His ECG on discharge was normal with QTc of 432 ms (Figure 3).

Presently, the patient is on his treatment, and on his request, he is referred to his native village. His regular ECG monitoring was done, and it was unremarkable.

## Discussion

DR-TB is considered to be a result of man-made problems due to spontaneous mutations in the genes of *Mycobacterium tuberculosis* and is a result of inappropriate/inadequate treatment [8,9]. The same is evident by the extent of MDR/RR-TB in new and previously treated cases, which is 3.6% (95% UI: 2.7%-4.4%) and 18% (95% UI: 11%-26%), respectively [3]. Cardiotoxicity is an ADR on the heart due to drugs such as bedaquiline, clofazimine, and moxifloxacin and needs immediate action as a delay in the management could prove fatal [9]. The commonest ADR of these drugs is prolonged QT intervals resulting in the acquired long QT syndrome (LQTS) with a proclivity to evolve syncope and unanticipated cardiac death owing to the malignant polymorphic ventricular arrhythmia known as torsades de pointes (TdP) [10].

The QT interval on an ECG is gauged from the initiation of the Q wave to the end of the T wave [10]. The normal values for QTc range from 350 to 450 ms and from 360 to 460 ms for adult males and females, respectively, with about 10%-20% variation in otherwise healthy individuals [11]. Ventricular conduction velocities and the velocity of repolarization have a direct impact on the QT interval [10]. A number of factors such as drug-drug interaction, blockade of ion channels, myocardium heterogeneity, electrolyte disturbances, and genetic polymorphism result in QT prolongation; nonetheless, the exact molecular mechanism of QT prolongation is unknown [10].

A case similar to this case was published by Kusmiati et al. (2021), where they reported three episodes of prolonged QTc interval in an elderly females, i.e., 545, 600, and 539 ms, respectively [12]. The present case differs from their case in age, gender, ethnicity, location, and type of treatment. Their case was given treatment with an injectable such as kanamycin even when bedaquiline was included in the regimens of most of the high-burden countries [12]. Further, the levels of QTc were not exceeding 688 ms, as reported in the present case. And there were no details of the efforts to rule out clofazimine as a causative agent, which has a moderate risk of QTc prolongation [12]. Besides, the case of Kusmiati et al. was restarted on moxifloxacin even after repeated episodes of QTc prolongation [12]. And the earliest episode of their case was on the seventh day, which is different from the sixth day as seen in this case.

In a study by Asfaw et al. (2021), only four (1.5%) out of 265 patients on bedaquiline- and/or delamanid-containing regimens developed high-grade QT interval prolongation [13]. In their study, they found four values of QTcF on the higher side, i.e., 523, 488, 518, and 536 ms [13]. In their study, clofazimine was found to be the likely cause in two cases and hypokalemia and borderline cardiac disease in one each [13]. The present case differs from their four cases with no involvement of clofazimine, hypokalemia, or borderline cardiac disease. Also, in their study, two cases were HIV-reactive, but the present case was HIV-non-reactive.

One study by Li et al. (2020) demonstrated that out of 120 cases, QTcF prolongation from baseline value of ≥60 ms occurred in 23 cases (19.2%) while QTcF of ≥500 ms occurred in 10 patients (8.3%) [14]. They inferred that the QTcF prolongation increased with the increase in bedaquiline exposure. They found that age of ≥45 years was a risk factor for QT interval prolongation; nevertheless, they didn’t report severe ventricular arrhythmia [14]. However, these findings were not noted in the present case.

Another study by Yoon et al. (2017), on 373 MDR-TB or nontuberculous mycobacterial cases, reported that a QTc prolongation was seen in 16.7% of cases [15]. Their maximum QTc value was 451 ms and a mean QTc lengthening of 33.6 ms from the baseline [15]. But the present case differs from their findings with QTc of 688 ms and QTcF of 638 ms.

In a randomized controlled trial on 84 subjects by Dooley et al. (2021), where either bedaquiline, delamanid, or both were added to a background regimen in patients with rifampicin-resistant TB, it was inferred that there was no more than a modest and not an additive effect on the QTc interval [16]. Additionally, there were no grade III or IV QTc prolongation events and no casualties during their study, and initial microbiology data at the eighth and 24th week were encouraging [16].

In short, this case was promptly diagnosed and managed due to the highly efficient national program for DR-TB control [6]. This patient could have suffered severe consequences if the treatment would have continued without the ECG monitoring. Furthermore, with the availability of newer drugs for TB control, it is imperative to train the field staff at the grassroots level for immediate management of any ADR. It is also recommended that this case report would serve as a basis for large-scale studies to draw conclusions regarding such abrupt changes in the QT interval. There are very few cases or studies reporting such QT interval abnormalities, and this paucity of literature is a limitation for the management of such encounters, especially in high TB burden settings.

## Conclusions

The management of multidrug-resistant TB is a challenging task especially due to the long treatment duration, pill burden, and ADRs. The situation becomes critical when the ADR could become fatal. The present case was such a case where ADR due to a very high QT prolongation with QTc of 688 ms was recorded on ECG, and with the immediate stopping of the cardiotoxic drugs, his baseline ECG was achieved with a QTc level of 432 ms. His treatment was restarted with the omission of moxifloxacin and the inclusion of delamanid. This case emphasizes the need for regular monitoring and supervision with regular training of all the field staff for any ADRs arising due to the use of multiple antitubercular drugs.
